# Suicide Prevention Measures at High-Risk Locations: A Goal-Directed Motivation Perspective

**DOI:** 10.3390/bs15081009

**Published:** 2025-07-25

**Authors:** Laura Joyner, Jay-Marie Mackenzie, Andy Willis, Penny Phillips, Bethany Cliffe, Ian Marsh, Elizabeth Pettersen, Keith Hawton, Lisa Marzano

**Affiliations:** 1 Department of Psychology, Middlesex University, London NW4 4BT, UK; 2 School of Social Sciences, University of Westminster, London W1W 6UW, UK; 3 NSPA Lived Experience Advisory Group, Ewell KT17 2AF, UK; 4 Faculty of Medicine, Health and Social Care, Canterbury Christ Church University, Canterbury CT1 1QU, UK; 5 Samaritans, Ewell KT17 2AF, UK; 6 Centre for Suicide Research, Department of Psychiatry, University of Oxford, Oxford OX3 7JX, UK

**Keywords:** suicide attempt, suicide prevention, goal pursuit, motivation, means restriction, built environment, surveillance, help-seeking

## Abstract

Understanding the effectiveness of suicide prevention measures for high-risk locations can often be challenging as many rely, at least to some degree, on psychological processes (e.g., engaging with help-seeking behaviours). Establishing how these measures may influence decision-making during a suicide attempt could be helpful for understanding how and when they may be most effective at preventing deaths. In the present work, we consider how suicide prevention measures may influence “goal pursuit” as it unfolds. Drawing on findings from across the suicide prevention literature, we apply the descriptive framework outlined in GOAL Architecture to consider how different measures may shape perceptions of “distance”, “time”, and “rate of progress” and, in turn, could influence levels of motivational drive associated with specific acts (e.g., “accessing means for suicide”). This is discussed in relation to real-time decisions around accessing means for suicide, avoiding intervention by a third party, and engaging in help-seeking behaviours. As well as the psychological processes that could encourage or prevent an individual from disengaging from a suicide attempt, we also consider potential risks and the influence of person-level factors.

## 1. Introduction

Annually, over 720,000 suicides are recorded globally (World Health Organisation, 2025). In the UK, around a third of suicides occur in public places such as railways, carparks, cliffs, and bridges (Office for National Statistics, 2023). Suicide has a lasting impact on families and friends, but suicides that occur in public locations can also create trauma for a wider group of people, including potential witnesses and staff dealing with their aftermath (Giupponi et al., 2019). They can also have substantial financial implications for affected industries, public health organisations, and responders (Holdaway et al., 2012). An additional concern about suicides in public locations is possible media reporting of incidents, which could highlight locations of concern and potentially lead to imitative behaviours (Marzano et al., 2025; World Health Organisation, 2023). Indeed, this explains why over 60% of local authorities in the UK currently implement interventions at high-risk locations, and why in England the National Institute for Health and Care Excellence (2018) recommends that suicide prevention measures are vital at high-risk locations.

Suicide prevention measures at high-risk locations are often categorised into four general approaches: (1) restricting access to means, (2) increasing opportunity and capacity for human intervention, (3) enabling help-seeking, and (4) changing public image of the site (Owens et al., 2015). However, understanding why certain measures have or have not been effective at preventing suicides is not always straightforward. For measures that fully restrict physical access to means for suicide, the explanation of how this can be effective is often clearer than for measures that rely, either to a degree or entirely, on psychological effects (e.g., signage encouraging help-seeking). If the processes underpinning such psychological effects are not considered, this arguably hinders the development of effective intervention design and creates barriers to appropriate evaluation. Exploring the influence of specific factors (e.g., measure placement, design characteristics) in a systematic manner may help to develop understanding around these psychological effects.

In the present paper, we draw from the psychological literature around goal pursuit, motivation, and behaviour with the aim of further understanding how, why, and when measures implemented at high-risk locations may prove effective at preventing suicides. Given the topic under discussion, it is worth noting that the use of formal, academic language to describe behavioural processes may not always align with the sensitivity needed to discuss subjects such as suicide. This is especially the case when key terms may also hold different meanings for wider audiences. To clarify, as we are interested in exploring the potential impact of suicide prevention interventions at high-risk locations on related goal-pursuit processes, the definition of a “goal” being “a cognitive representation of a desired end state that a person is committed to attain” (Milyavskaya & Werner, 2018, p. 5) applies here. For the purpose of this paper, we therefore view a goal to be something that is specific or abstract that an individual wants to approach or avoid in the future that may shape their decisions and ultimately guide their behaviour. The term “motivation” is also used to reflect “a person’s willingness to exert physical or mental effort in pursuit of a goal or outcome” (American Psychological Association, n.d.). Our use of this term refers specifically to the amount of motivational drive associated with individual goals (i.e., “motivational value”).

While it may seem counterintuitive to view suicide through a lens of “goal attainment”, research on goal pursuit has provided the basis of much work on behaviour change, including within health psychology (e.g., Schrooten et al., 2012). When a goal becomes activated, an individual may mobilise resources and identify actions for pursuit. However, both the goal and these underlying processes may occur unconsciously, i.e., without the person being actively aware this is happening (Kopetz et al., 2018). Yet, it is in no way suggested here that decisions and evaluations made when individuals are under mental distress and/or in response to adverse events and circumstances are always conscious.

Throughout this paper, we consider a small selection of specific goals that may or may not be active while an individual is approaching or reaches a high-risk location (i.e., “access area of danger”, “avoid intervention by third party”, “seek help”), rather than looking at suicidal behaviours more broadly. We believe it is helpful to focus on these specific examples set within what may be a relatively brief moment in time (vs. a person’s lifetime) to further our understanding of the psychological effects associated with different types of suicide prevention measures that may be deployed at high-risk locations specifically and to inform ways of quantifying their effectiveness. While this means that the present work does not address the periods leading up to or following an attempt, prior research looking at goal pursuit in the context of self-injurious and other high-risk behaviours suggests that people may be less likely to engage with such behaviours when alternative means to fulfil their needs (e.g., self-regulation of emotions) are provided (Kopetz et al., 2018). While not the main focus of this article, the wider systemic issues that may present barriers for some individuals to fulfil their needs (e.g., accessing help) are therefore worth considering in this context. Examples of this are discussed here in the context of help-seeking (e.g., prior barriers to access).

Before exploring the relationship between specific suicide prevention measures and attempt-related actions in more detail, we look first at existing work from the suicide prevention literature that has addressed issues relating to “goals” and “motivations”. Next, we provide some wider background on processes of goal prioritisation and dynamic changes to motivation. We then explore how measures recommended for high-risk locations may influence levels of motivational drive from a goal-directed motivation perspective—specifically, how the use of measures that restrict access to means, that increase the likelihood of third-party intervention, and that encourage help-seeking, may change the path of goal pursuit along the gradients outlined in GOAL Architecture (i.e., rate of progress, distance, time).

### 1.1. Goal Pursuit & Suicide

Across the suicide prevention literature, the concepts of “goals” and “motivation” have been researched in varying ways. Firstly, a body of work has looked at the motivations associated with suicide attempts, such as hopelessness or burdensomeness (e.g., May et al., 2020; May & Klonsky, 2013). Others have considered suicide risk in the context of goal regulation processes, such as goal re-engagement and disengagement (e.g., O’Connor et al., 2012). Baston (2024) applied a goal system approach to help explain suicidal behaviour. In addition, there is work applying action theory to describe suicidal behaviour as a goal-directed process (e.g., Valach et al., 2006, 2016). While this body of work is useful for developing an understanding of the psychological processes preceding a suicide attempt, less is known about decision-making processes during the attempt itself.

An individual holds multiple goals organised across different levels, some of which may be complementary, potentially even facilitating subsequent or overarching, superordinate goals. From this perspective, an individual who holds an abstract superordinate goal related to “making a suicide attempt” could pursue this goal via several concrete subordinate goals. In turn, these subordinate goals may reflect specific, sequential actions that could contribute towards an attempt (e.g., “access a high-risk area”) or support the pursuit of this goal in other ways (e.g., “avoid interventions by other people”). While previous research on suicide prevention and goals has focused more on the superordinate level (e.g., identifying underlying motives), in interviews individuals with lived experience of surviving a suicide attempt have talked about “the steps” involved (Marcinkevičiūtė et al., 2024), suggesting the subordinate level is also relevant.

It is also notable that in both research and guidance, suicide prevention measures for high-risk locations are often categorised in ways that relate to subordinate-level goals—for example, “prevent access to means”, “increase opportunity for human intervention”, or “encourage help seeking” (e.g., Owens et al., 2015). Exploring the ways in which suicide prevention measures may then influence the “micro-decisions” made by individuals (e.g., approach, retreat, seek out alternate routes, etc.) in relation to these actions could arguably help further our understanding of how and when they prove most effective at preventing suicides, or even identify when they may risk causing harm.

Moreover, the principles of goal-directed behaviour may still apply when individuals are experiencing emotional distress. Kopetz and Orehek (2015) suggest that physical self-harm produces immediate consequences that aid the attainment of emotional regulation goals. For example, previous work found the application of bodily harm (vs. a sham condition) resulted in reduced stress responses in participants who had engaged in non-suicidal self-injury (Reitz et al., 2015). Put simply, rather than a failure of self-regulation, Kopetz and Orehek (2015) propose that behaviours such as self-harm may allow some individuals to achieve an immediate goal of regulating emotional distress. Importantly, however, such self-harming behaviours may also be reduced if alternative means of fulfilling the goal are provided. This body of work not only challenges the idea that emotional distress impairs cognitive control but also illustrates how potentially harmful actions may be pursued in the moment if perceived to be an effective way of addressing immediate needs (especially if safer alternatives are not accessible).

We believe that such an approach is relevant for a number of other reasons. Firstly, viewing goal pursuit as a complex, dynamic process allows for the possibility that individuals hold a broad variety of goals in the lead-up to and during a suicide attempt. Indeed, the goal-directed motivation literature suggests individuals may be unable to pursue all their goals at any one time (and therefore must prioritise specific goals over others). Considering which goals may facilitate an attempt and therefore may be activated (e.g., avoid attention, avoid third-party intervention) and which conflict and therefore may become inhibited (e.g., social or help-seeking goals) may be useful for understanding the effectiveness of specific measures, particularly when used in combination with other interventions.

Moreover, understanding how these measures may themselves shift the prioritisation and pursuit of subordinate-level goals could prove more valuable in understanding their effectiveness than simply focusing on the superordinate (e.g., overarching attempt-related goal). In particular, distinguishing between the expected impact of a suicide prevention measure on the pursuit of a specific action, independent of the reasons behind the presentation, could be a useful step towards understanding their effectiveness across a range of different contexts. This aligns with certain general theories of behaviour—for example, the Theory of Interpersonal Behaviour, where “facilitating conditions” moderates the relationship between “intentions” and “behaviour” (Triandis, 1977). From this perspective, by influencing the situational context, suicide prevention measures have the potential to inform decision-making around goal pursuit in real time, but only to a point (e.g., disengaging from an “access means for suicide goal” at one location does not necessarily mean a person will also disengage from the attempt generally). Such an approach allows us to consider the influence of variations in strength of motivation (i.e., motivational drive) and other person-level factors (e.g., self-efficacy), which, as we discuss throughout the paper, may help those working in suicide prevention distinguish when and how measures relying on psychological effects are most effective.

#### Goal Prioritisation

Goal prioritisation is a dynamic process allowing us to navigate complex environments via constant decisions around “which goals to prioritise, how much to invest in pursuit of those goals, and when to divert their attention towards other goals” (Ballard et al., 2022, p. 146). While decisions around goal prioritisation may sometimes involve deliberative thinking, research suggests that they can also be made intuitively—for instance, by opting for heuristic strategies when time or cognitive resources are more limited (Alister et al., 2024). Notably, dual-process theories of cognition suggest that such intuitive decisions occur rapidly, are automatic, and at times are made unconsciously (Evans, 2008). This suggests that choices around the prioritisation of which goals to pursue are not always based around effortful decision-making processes based on logic or critical thought. Instead, situational cues may influence choices around whether a person continues to pursue or disengage from a specific goal in ways that do not require effortful deliberation or even conscious thought.

An important factor in goal prioritisation is the goal’s motivational value, as in, “the extent to which one is driven to allocate resources to it” (Ballard et al., 2022, p. 146). This drive may firstly be influenced by desire, which is “an affectively charged motivation toward a certain object, person, or activity that is associated with pleasure or relief from displeasure” (Hofmann & Van Dillen, 2012, p. 317). In some instances, desire may correspond with a specific goal and therefore provide varying degrees of motivation towards goal pursuit. In other contexts, desire may conflict with a goal and instead present a temptation to deviate away from said goal, requiring an individual to engage with effortful self-regulation to counter its effect (Hofmann & Van Dillen, 2012). Desire is therefore an important consideration when seeking to understand the pursuit of goals related to suicide attempts, as research suggests suicidal crises may be motivated by strong affective states such as extreme emotional distress (e.g., May et al., 2020; May & Klonsky, 2013). As such, a person may be motivated to pursue a potentially harmful goal (e.g., a suicide attempt) if they associate it with their desires (e.g., a drive to relieve emotional distress and/or feelings of entrapment). If that is the case, the strength of this desire will then also influence how motivated individuals are to pursue such a goal.

**Figure 1:**
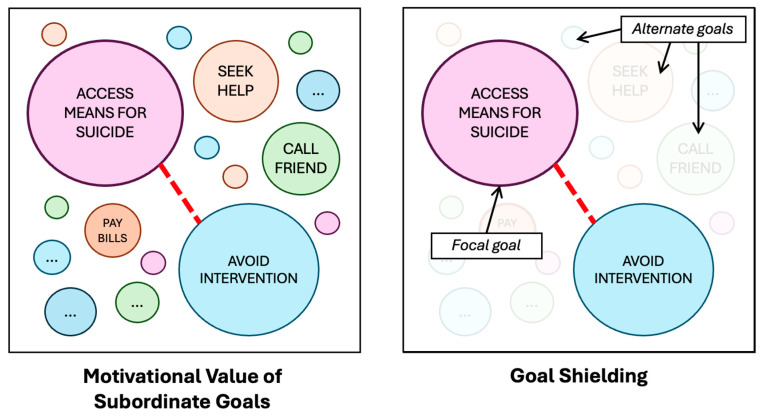
Examples of goal-directed motivation and the goal-shielding process.

While the decision-making processes involved when pursuing a goal may at times be intuitive and even unconscious, they can still inform behaviour (e.g., taking a familiar route). Motivational value may also influence behaviour—for example how much effort an individual is willing to put in, their willingness to persist when faced with obstacles, and specific task-related choices (e.g., if motivated to avoid intervention, a person may turn left if there is a police officer to their right). As illustrated in Figure 1, when an individual is beginning to pursue specific tasks or “steps” leading up to a suicide (e.g., travelling to a location, accessing means, avoiding other people/intervention), the strength of motivation driving those steps may be relatively higher than that for alternative goals (e.g., help-seeking). Moreover, research also suggests that when an individual is highly committed to a “focal goal”, alternative goals may be cognitively inhibited (Shah et al., 2002). In other contexts, this automatic and unconscious cognitive process, known as “goal shielding”, may be greatly beneficial for helping reduce external distractions that may hinder successful goal attainment. However, given that goal shielding can also be influenced by factors including emotional state (Plessow et al., 2011; Shah et al., 2002), such cognitive processes are likely to also be important considerations for understanding how and when some interventions can prove effective (e.g., help-seeking signage).

The motivational value of any goal also changes over time. On one hand, this may relate to changes to the perceived value of the goal—for instance, how desirable achieving the goal is. However, the motivational value may also be influenced by feasibility—as in, how likely it is the goal will be achieved (Milyavskaya & Werner, 2018). Feasibility may be shaped by a broad range of external factors that may facilitate or, alternatively, threaten goal attainment. For instance, an individual with a goal to have a healthy lunch at work (a subordinate goal relating to a broader, superordinate health goal) may feel less motivated to pursue this specific goal if the store they intended to purchase lunch from is considerably farther away than unhealthier alternatives. Furthermore, while distance alone might not deter an individual from pursuing their healthy lunch goal, if colleagues were then to invite them to lunch at a nearer restaurant, this relative closeness may provide added motivation required to prioritise a different superordinate goal, e.g., one related to social needs. As a result, they may no longer pursue the original goal of visiting the shop for healthy food but instead adjust (e.g., selecting a healthy menu option) or even abandon the healthy eating goal for that day. While it may be distant from the issue of suicide, this example illustrates how the dynamic nature of goals allows us to navigate complex physical and social environments.

**Figure 2:**
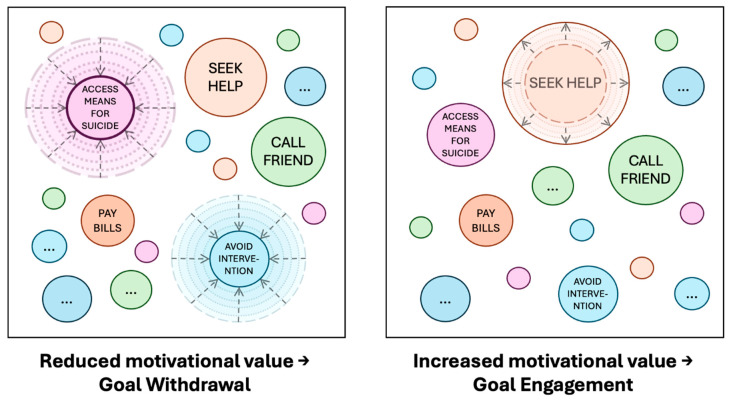
Goal re-prioritisation through changes to motivational value.

In the context of suicide prevention at high-risk locations, it may be that specific preventative measures and other related factors (e.g., the presence of members of the public) at times threaten the perceived feasibility of achieving attempt-related goals in ways that influence their motivational value (Figure 2). If that is the case, then factors within an encountered situation (e.g., means restriction measures, presence of others) may encourage an individual to re-evaluate feasibility and in turn could result in a reduction in the motivational value of the specific goal. At the same time, alternative goals that may previously have been inhibited (e.g., help-seeking) may become more salient, especially if changed conditions make this more feasible or attractive. From this perspective, situational factors could influence the prioritisation of goals in real time in ways that could aid (or, in other cases, potentially undermine) suicide prevention efforts.

Recently, a descriptive framework has been proposed to help quantify these motivational dynamics. The Goal-Orientated Action Linking (GOAL) framework integrates key theories of goal pursuit to help quantify dynamic changes in goal prioritisation (Ballard et al., 2022). Ballard et al. (2022) also note that the framework can be used to better understand the motivational dynamics that occur when pursuing a goal, e.g., across interdependent decisions. This may therefore be particularly relevant when trying to understand the psychological impact of encountering multiple suicide prevention measures at a high-risk location. The framework assumes that the perceived impact of one’s actions on goal attainment influences levels of motivation towards achieving one’s goal and suggests that motivational value varies and updates in response to three gradients: distance (i.e., proximity), time to deadline, and rate of progress required (i.e., how hard the goal is to achieve). These gradients then provide a basis for quantifying the extent to which an individual may be driven to allocate resources to achieve a particular goal. Throughout this paper, we will therefore consider the potential impact of suicide prevention interventions and other relevant factors on these gradients to understand how they may impact motivation and, in turn, influence behaviour.

## 2. Restricting Access to Means for Suicide

While arguably not as effective as “full” restriction, research suggests implementing partial restriction to means may help to prevent suicides. These measures do not entirely fully restrict access to an area—for instance, due to barriers or other measures preventing access being lower or not long enough to cover all possible access points (Shin et al., 2024). Despite this, there are examples of partial restriction measures that have contributed to an overall reduction in suicides on railways (e.g., Fredin-Knutzén et al., 2022), at bridges (e.g., Shin et al., 2024), and at high-rise buildings (Mohl et al., 2012), as well as examples where they did not (e.g., Hemmer et al., 2017; Shin et al., 2024). It is therefore important to determine how and why these mitigations work in preventing suicides to better inform intervention design in the future.

As previously discussed, the application of the framework from GOAL Architecture (Ballard et al., 2022) may provide a useful way of interpreting and quantifying the effects of measures that restrict access to means. While certain “full restriction” measures will, of course, present a substantial physical barrier to access, it is sometimes suggested that partial restriction measures act more as a psychological deterrent (e.g., Ross et al., 2020). This is particularly the case where physical access is in no way restricted—for instance, when vehicle (but not pedestrian) access to a high-risk location is restricted (Isaac & Bennett, 2005; Skegg & Herbison, 2009). Yet, research also indicates that not all physical adjustments within an environment will have an impact on suicide rates, suggesting that there are nuances to how and when any psychological deterrent effect occurs. The following section therefore explores how two gradients from GOAL Architecture could be applied to better understand the psychological effects of means restriction measures. First, we discuss how changes to perceived access may influence the effectiveness of some means restriction measures, and how effectiveness may differ across population groups. We then discuss the influence of proximity on motivation to access a high-risk area, both in the context of measures that increase proximity and of measures that cover only some access points.

### 2.1. Rate-of-Progress Gradient

The Interpersonal–Psychological Theory of Suicide (Van Orden et al., 2010) identifies acquired capability as an important factor for understanding suicide attempts. Experiences such as mental rehearsal, “dry runs”, and familiarity (as well as temporary states such as intoxication) may influence perceived capability (Smith & Cukrowicz, 2010). In the context of means restriction, individuals with lived experience of surviving suicide attempts have also indicated that ease of access may be an important decision-making consideration when looking to access a high-risk area (Mackenzie et al., 2025; Marsh et al., 2021; Marzano et al., 2021; Norman et al., 2023; Rosen, 1975). From a goal-pursuit perspective, it is suggested that a “person is motivated to prioritise a goal to the extent that doing so increases their chances of attaining the goal and/or failing to do so decreases their chances” (Ballard et al., 2022, p. 167). As such, a person’s beliefs about the likelihood of progressing past full or partial measures (i.e., their outcome expectations) may provide some insight into understanding why and when measures may prove effective at preventing suicides.

Across the goal-pursuit literature, there are different perspectives as to how “rate of progress” may influence motivational value (Ballard et al., 2022). There is the Expectancy Perspective, which suggests that a goal’s motivational value will be greater when it is easier to achieve. However, in some contexts, some people may be motivated by challenging goals (the difficulty perspective). Another approach, the achievability perspective, suggests “motivation is the highest when the rate of progress required to reach a desired or undesired state is moderate” (Ballard et al., 2022, p. 151). Depending on the task at hand, there is evidence for all three perspectives. As person-level factors such as individual differences and skill level can also influence the way in which perceived effort impacts motivation (Ballard et al., 2022), considering how the design of measures may increase difficulty–ease of access may also be useful for understanding context-specific effectiveness.

#### 2.1.1. Environmental Changes to Decrease Ease of Access

For some, the potential benefit of applying such a psychological framework to better understand why full means restriction can help prevent suicides may be unclear. Arguably, some measures will simply make the prospect of proceeding past them too physically challenging for most people. For instance, research suggests that barriers above 2.3 m are more effective at preventing suicides on bridges (e.g., Hemmer et al., 2017). Similarly, studies that have investigated the impact of removing restriction measures have found this to be associated with an increase in deaths by suicide at some high-risk locations (Beautrais, 2001; Beautrais et al., 2009). Such examples illustrate how making access to means extremely difficult or at times impossible can help prevent suicides. However, at many high-risk locations, full restrictions to means may not always be possible or even feasible. For example, engineering constraints such as weight restrictions may prevent the installation of new barriers on some bridges. Alternatively, at railway stations that serve different models of trains, variations in door positions may prevent the installation of platform-screen doors. Measures that only partially restrict access to means may therefore be a more viable alternative in some contexts.

While little is known about the psychological mechanisms underpinning any preventative effects of partial restriction measures, researchers have previously suggested that these measures may reduce the perceived ease of access (Fredin-Knutzén et al., 2022; Mohl et al., 2012) or make access more difficult (Mackenzie et al., 2025; Shin et al., 2024). Furthermore, in a previous qualitative study, one individual with lived experience of surviving a suicide attempt indicated that upon encountering a fence that they felt in another context they would be able to climb, the substantial effort to access the high-risk area (e.g., by climbing over) produced doubts that then influenced their decision not to proceed further (Heesen et al., 2024). This might suggest that while an individual’s judgements around perceived difficulty may not directly influence any overarching (superordinate) suicide-related goal, they may instead allow a specific attempt-related act (subordinate goal, e.g., access a high-risk area) to be deprioritised in the moment.

Evaluating the literature from this perspective may therefore help provide insights into how, why, and when certain measures prove effective at preventing suicides. Firstly, the height of a barrier provides a clear quantifiable example of how a means restriction measure may impact perceived difficulty. For example, “full” barriers of heights of 5 m (Sinyor & Levitt, 2010), over 3 m (Dwyer et al., 2023; Hemmer et al., 2017; Pelletier, 2007), and over 2.3 m (Hemmer et al., 2017) have previously been associated with complete reductions in suicide rates at bridge locations. Here, the height of these measures alone suggests it is likely difficult for most people to scale them unassisted (although characteristics related to materials and design may play a further role (e.g., Dwyer et al., 2023)).

Moreover, barriers around 1.9 m to 2 m (Bennewith et al., 2007; Hemmer et al., 2017) have been associated with significant, but not total, reductions in suicide rates on bridges (notably, such barriers are below recommended minimums, e.g., 2.5 m, as recommended by Owens et al. (2015)). Learnings around the effectiveness of even lower height barriers may provide important insights into when and how means restrictions measures prove effective at preventing suicides.

**Figure 3:**
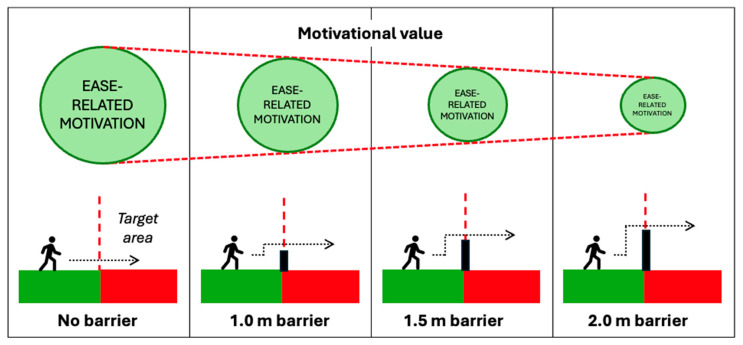
Example of the influence of increasing barrier height on motivational value.

Indeed, research suggests that compared to no barriers, lower barriers prevent some (but not all) suicides. For example, installing “half-height platform screen doors” 1.2 m–1.65 m high has previously been associated with a significant, but not complete, reduction in suicide rates on metro platforms (Chung et al., 2016; Okada & Oshima, 2025; Ueda et al., 2015; Xing et al., 2019). In another study, the addition of 1 m-high mid-track fencing (i.e., intermediate fencing between tracks restricting access to high-speed lines) was associated with a reduction in suicides at a railway station (Fredin-Knutzén et al., 2022). An important point to note here is that in both types of environments there previously would have been no physical barrier to entry. This is arguably very different from interventions adding further height to a bridge barrier, for instance, where it is likely that some form of barrier (albeit a smaller one) was already in place. Similarly, previous work suggests that differences in height of as little as 30 cm (i.e., 1.2 m vs. 1.5 m) may influence the level of effectiveness of barriers (Xing et al., 2019). As illustrated in Figure 3, as the height of barriers increases, the perceive ease of access may decrease, which, in turn, may have an additive influence on reducing motivation.

**Figure 4:**
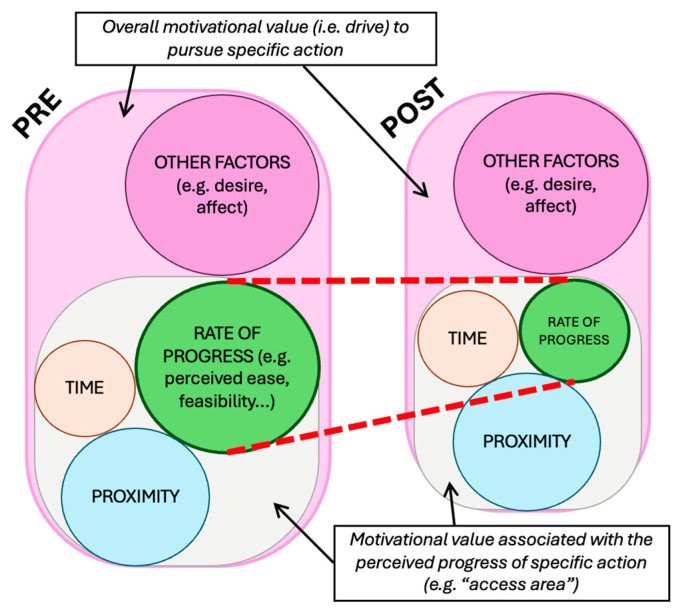
Influence of gradient-related changes to motivation on a goal’s total motivational value.

While the extent to which any prevention effects observed with smaller or lower barriers are due to physical factors is unclear, others have suggested barriers of similar heights are not necessarily considered difficult to climb (Ross et al., 2020). As Ross et al. (2020) also suggest, such measures may act as psychological deterrents, at least for some individuals. Indeed, from a goal-pursuit perspective, the presence of partial barriers may influence the perceived ease or difficulty of progressing further (e.g., to reach an area of danger and/or to do so as inconspicuously as possible). If that is the case, then the presence of such measures may prompt individuals to re-evaluate and potentially update their beliefs about the likelihood they will be able (and the effort required) to proceed onwards (i.e., outcome expectations). Where a measure adds a (perhaps unexpected) degree of difficulty, re-evaluation of outcome expectations may, at the very least, prompt hesitation (e.g., Heesen et al., 2024). It may also have the potential to influence the total motivational value of the goal itself (Figure 4). If changes to the perceived rate of progress are substantial enough, for some individuals this could be enough to encourage withdrawal from the goal (at least in the immediate term). This might provide one explanation as to why lower barriers are effective for preventing some, but not all, suicides.

Beyond adjustments to the height of barriers, considering how means restriction measures relate to one another across an ease–difficulty spectrum may be useful for developing a better understanding as to when and why they do (or do not) help prevent suicides. The potential value of this approach perhaps becomes clearer when considering other types of physical adaptations to an environment that have demonstrated some benefits in the context of suicide prevention. For example, research suggests there may be other types of modifications that could also be helpful at preventing suicides by making access more challenging. For example, at bridges, the use of curved barriers (Beautrais et al., 2009; Hemmer et al., 2017; Perron et al., 2013) and rolling bars (Shin et al., 2024) has been associated with reductions in suicides. Such modifications that increase the difficulty of accessing a space in ways other than simply increasing height are also recommended in official guidance (e.g., Owens et al., 2015). However, while factors like changes to height can easily be measured and compared, without formal frameworks to help quantify the psychological impact of other types of changes influencing perceived ease of access, it remains difficult to make formal comparisons. This is where considering the impact of physical adaptations on perceived ease–difficulty of access could be useful for understanding how means restriction measures work, as both physical and psychological deterrents.

Finally, interpreting the influence of a measure in relation to perceived ease–difficulty may also be useful to understand why certain interventions fail to be effective “psychological deterrents” when others appear to prevent at least some deaths. For example, Mohl et al. (2012) found that the placement of a single guard rail bar 18 cm above windowsills reduced suicides from a high-rise hospital building. Yet, while this minimal adjustment to an environment was found to be effective, another study found that the addition of lightweight, 10 cm-tall bird spikes to a 90 cm bridge parapet was not effective at preventing suicides (Shin et al., 2024). On one hand, it may be that the findings observed here are in relation to their impact on barrier height. However, from a goal-pursuit perspective, it could also be that relatively weaker and potentially easier-to-remove mitigations do not present a substantial-enough barrier to impact motivation. Indeed, interviews with individuals with lived experience of surviving a suicide attempt indicate that barriers left in poor condition may be seen as potentially breakable, which in turn can influence the perceived ease of access (Mackenzie et al., 2025). Together, this suggests there may also be a need for measures to influence perceived difficulty in a meaningful way—not just in terms of height, but also in terms of factors such as physical robustness, materials, and surface texture playing a role. For example, industry research has identified some of the strengths and weaknesses of physical attributes for a range of fencing designs, including factors such as ease/difficulty to scale, but also cutting with tools (Stanchak et al., 2015). Distinguishing and quantifying specific design features across an ease–difficulty spectrum may therefore be beneficial to help further develop our understanding of effectiveness of measures across a variety of factors.

#### 2.1.2. Integrating Person-Level Factors

As previously noted, certain means restriction interventions may be effective at preventing some but not all deaths by suicide. This means there is arguably value in understanding for whom the measures are or are not effective, and for determining why this may be the case. From a psychological perspective, it is not just environmental factors that influence behaviour but also person-level factors. This can include factors such as overall motivational desire and affective states, both arguably important factors in understanding suicidal behaviour. However, Social–Cognitive Theory (SCT) suggests that both person-level and environmental-level factors can interact with one another in ways that influence behavioural outcomes (e.g., Schunk & Usher, 2012). Similarly, the Theory of Interpersonal Behaviour suggests the level to which conditions “facilitate” behaviour moderates the relationship between intentions (e.g., affect) and behaviour (Triandis, 1977). Put simply, the extent to which a physical measure is effective in encouraging an individual to disengage from proceeding onwards into a high-risk area is likely to vary in relation to person-level factors.

An example of this is where the level of effectiveness of interventions differs across genders. In one study, a downwards trend in suicides was found only for women after a combination of measures was introduced (including a 1.3 m fence) at a high-risk location (Ross et al., 2020). As previously noted, focus groups with police indicated that the fence itself was not difficult to climb, and the authors therefore suggested the fence may have presented more of a psychological barrier than a physical one. In another study, it was found that only 1 out of 10 people who died after climbing over a half-height platform screen door were women (Xing et al., 2019). While it is of course not possible to rule out differences in physical ability, this suggests there may be potential value in developing a more detailed understanding of the potential impact of person-level factors on intervention effectiveness. For example, understanding when factors such as gender or age influence the effectiveness of certain measures may be useful for informing the development of safer measures in the future.

Additionally, self-efficacy may influence the effectiveness of some means restriction measures if decisions are based on a perceived reality that, despite feeling “true”, may not be factually accurate (e.g., about own strength). If beliefs around perceived difficulty and outcome expectations are biased, this could lead an individual to proceed in a situation where others with the same physical ability might refrain. Were an individual to overestimate their ability (e.g., for climbing), their motivation to proceed onwards may not be affected in the way that it might be for others with lower self-efficacy beliefs. This may be particularly important to consider given what is known about who may be at increased risk of suicide. For instance, during a psychotic episode, some individuals may perceive themselves to have special abilities, such as heightened strength (Ritunnano et al., 2022), which could influence their sense of self-efficacy.

Another consideration is where an individual may have challenges around evaluating the likelihood of a negative outcome. For example, individuals at greater risk of experiencing hypomania may perceive the consequences of risk-taking to be less costly (Devlin et al., 2015), which may mean that the requirement to apply additional effort to access a high-risk area may present as less of a psychological deterrent for such individuals compared to others. While research is required to better understand the impact of individual differences on intervention effectiveness via changes to perceived difficulty, together, these examples illustrate the possibility that “psychological deterrent” effects may not produce similar outcomes across the wider population. If it is the case that such effects are influenced by individual differences, adjustments to design standards may be needed to ensure preventative effects occur across all relevant populations. This is particularly important for ensuring both the effective and equitable development and deployment of future suicide prevention initiatives.

Finally, whether the level of effort required to pass means restriction measures is anticipated may be important for understanding their effectiveness as psychological deterrents. For instance, a person may approach an environment having anticipated no substantial barriers to entry and therefore believed the perceived likelihood of being able to proceed to be relatively high. If they were then to unexpectedly encounter, for instance, a fence, this may prompt an individual to re-assess their beliefs around the likelihood of proceeding further. From a goal-directed motivation perspective, if the perceived rate of progress (e.g., level of difficulty) increases enough to change the outcome expectation, then this may lead to a reduction in the motivational value of the related goal. If this reduction were great enough, then an individual may re-evaluate, and potentially disengage from, pursuing the specific act further (at least temporarily). One consideration might therefore be how prepared an individual is to anticipate the level of effort required to proceed into a high-risk area. Where individuals attend a location with an accurate expectation of the effort required to access a high-risk area—for example, due to planning (e.g., Mackenzie et al., 2025; Marsh et al., 2021)—they may be less likely to need to update these beliefs. The influence of accrued knowledge about a location on the context-specific effectiveness of suicide prevention is therefore another potentially important person-level factor to consider.

### 2.2. Distance Gradient

**Figure 5:**
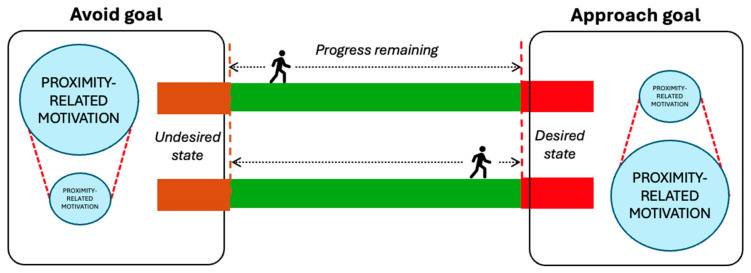
Influence of proximity on motivational value for avoid and approach goals.

Another important aspect identified in the framework proposed by GOAL Architecture is the influence of remaining progress (e.g., distance) on motivation. This is because previous work suggests that motivation increases when the target of the goal is closer (Ballard et al., 2022). The influence of distance on motivation operates in two ways. Firstly, when a person is seeking to avoid an undesired state, the motivation to proceed onwards would likely decrease as they get further away from the target. For at least some suicidal individuals, this may mean that increased distance from, for example, a triggering event may gradually reduce motivational drive. Indeed, previous work has identified elevated risk of suicide in the first six months following a cancer diagnosis (Henson et al., 2019) and in the first 30 days following a bereavement (Pitman et al., 2023). In contrast, when a person is approaching a desired goal (e.g., accessing a high-risk area), their motivation to proceed onwards may increase the closer the target becomes (Figure 5). For example, in relation to rail suicides and suicide clusters, several studies have found that most deaths occur in close proximity to the deceased’s home address (e.g., Mishara & Bardon, 2016; Norman et al., 2024; Too et al., 2017). Combined, this illustrates the complex, dynamic nature of goals, as well as the potential impact of distance/proximity in the context of suicide prevention.

In this section, we consider how distance (more specifically, perceived distance remaining) may also influence the effectiveness of means restriction measures. First is the way in which the physical placement of measures may influence their effectiveness. Next, we consider examples where measure design may have not sufficiently increased perceived distance (e.g., through gaps in coverage).

#### 2.2.1. Placement of Intervention

As previously discussed, if a person’s goal is to access a specific location (i.e., an approach goal) any “distance-related motivation” may be strongest when there is little further progress remaining. This might suggest that changes that increase the perceived progress required to access means could reduce motivation in ways that could produce a deterrent effect. Indeed, research suggests that introducing restrictions to the quantity of drugs such as paracetamol allowed in a single purchase can help reduce the number (as well as size) of overdoses with these drugs in the population (Hawton et al., 2001). Arguably, such restrictions do not prevent an individual from accessing means but instead increase the amount of effort required to do so (e.g., implementing a need to make multiple purchases across different locations and/or times), which should reduce motivation to proceed onwards. While this, of course, may not result in all suicides by this method being prevented, it provides one potential explanation for the psychological processes associated with this approach.

There is also evidence to suggest that “distance remaining” may be important in the context of high-risk locations. Previous work suggests that proximity to a high-risk area may influence the strength of suicidal thoughts (Marsh et al., 2021) or increase urges (Norman et al., 2023). Individuals have also reported increasing distance (e.g., stepping back) from a high-risk area to self-regulate their behaviour (Marsh et al., 2021). As such, adjustments to perceived proximity (e.g., via changes to physical distance or steps required) may be another relevant (and quantifiable) factor for understanding the effectiveness of suicide prevention measures from a psychological perspective. Indeed, studies suggest that the placement of safety nets placed underneath bridges may be effective at preventing some suicides (Hemmer et al., 2017; Reisch & Michel, 2005; Shin et al., 2025). This is a measure that arguably cannot prevent a person from proceeding beyond the net but does introduce an additional layer to access (i.e., increases progress required) that potentially also creates uncertainty around outcome expectations.

**Figure 6:**
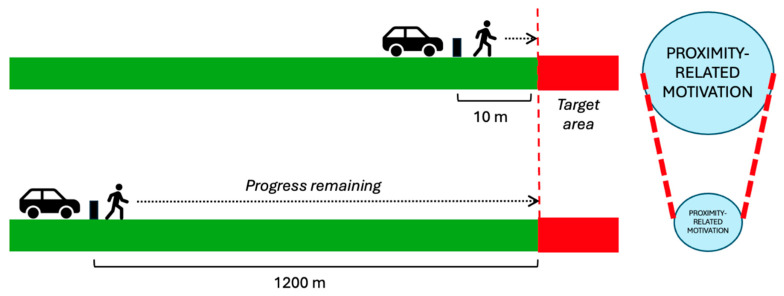
Example of the impact of vehicle restriction measures on perceived proximity and associated motivational value.

The potential influence of perceived progress on motivation may also help provide insight into why restricting vehicle access to a high-risk location while retaining pedestrian access has previously been associated with reductions in deaths by suicide (Isaac & Bennett, 2005; Skegg & Herbison, 2009). While both sites remained accessible to pedestrians, these temporary road closures meant that the distance required to travel on foot (e.g., vs. from a closer car park) increased during these periods, which were 5 months and 2 years, respectively. For instance, in the study by Skegg and Herbison (2009) a 1.2 km–1.6 km length of road approaching a cliff lookout was closed to vehicles. At both locations, no deaths were recorded during the road closure periods. These findings are particularly notable examples of the dynamic nature of goal value. While in these examples the true physical distance to access a high-risk area did not change, the overall progress required to cover the same ground on foot was greater compared to when a vehicle was used. Arguably, in many cases, the preventative effect observed in these studies would therefore primarily be psychological, as individuals themselves were not prohibited from entering either site (only vehicles). What is not known is whether smaller distances of vehicle restriction would have the same effect. However, from a goal-pursuit perspective, the physical proximity of the placement of such measures would likely be an influencing factor (Figure 6). If the motivational value of approach goals increases as a goal’s reference point becomes closer, then this kind of intervention may be more effective the farther away they are positioned. As we discuss in the next section, smaller distances may not be as successful for preventing suicides. However, future research is needed to understand whether the proximity of intervention placements does indeed influence effectiveness for suicide prevention as the goal-directed motivations literature might suggest.

#### 2.2.2. Gaps in Coverage

Another way in which distance may influence the effectiveness of means restriction measures is the proximity of alternative access points. For example, a previous study found that fencing implemented across railways was only associated with a reduction in suicide rates at sites where fencing was over 100 m in length (Clapperton et al., 2023). Specifically, there was a 57% reduction in suicide rates within a 1000 m radius from the midpoint of fences 100 m or greater in length. In contrast, sites with shorter stretches of fencing saw no significant reduction in suicides. This could suggest that some people may have been dissuaded from travelling along the fence to find an access point (i.e., where the fencing ends), but only where the distance required to travel is great enough.

Similarly, previous research focusing on bridge sites found barrier-related measures that only covered sections of the bridge (e.g., otherwise leaving gaps for entry elsewhere) were not effective at preventing suicides (Sæheim et al., 2017; Shin et al., 2024). Bridges where barriers have only been added to some (but not all) areas have also observed patterns of localised displacement to other parts of the bridge after installation, albeit with some reduction in suicides (Bennewith et al., 2007). Conversely, removing gaps as part of wider improvements to the design of safety barriers (Beautrais et al., 2009) or through the removal of level crossings (Clapperton et al., 2022) has been associated with a reduction in suicides. Together, these findings suggest that smaller adjustments to perceived progress remaining may not be enough of a deterrent to encourage goal withdrawal, particularly if motivational value (e.g., due to drive) is already relatively high.

## 3. Increasing Likelihood of Third-Party Intervention

Where an environment is in some way monitored, either formally (e.g., by camera surveillance) or through the presence of other people (e.g., members of the public), it can increase the likelihood that an intervention by a third party may occur. Therefore, while the underlying reasons for a person looking to avoid an intervention may vary (e.g., wanting to avoid police intervention), a common factor is that attention from others may threaten their ability to achieve an “avoid = third-party intervention” goal as well as any associated goals (e.g., “access means”). In this section, we propose that the perceived risk of intervention influences behavioural motivation via changes to the time gradient. In other words, believing that an intervention may occur potentially creates perceived timeframes within which intended actions seem attainable. For example, in a busy environment with overt surveillance (e.g., cameras, patrols) the perceived window of time to “act” without being noticed may potentially be much shorter than in a quiet environment with no visible surveillance. This would have important implications from a goal-pursuit perspective, as motivation can increase when there is a looming deadline, and time available can also influence perceived feasibility, e.g., the likelihood of achieving a goal in the remaining time (Ballard et al., 2022).

Using the existing suicide prevention literature, we first discuss how the presence of other people and surveillance may influence behaviours leading up to a suicide attempt. We then consider the influence of suicide prevention measures on perceived visibility and behaviours. Finally, we discuss how measures can be combined to highlight “atypical behaviours” in ways that indirectly influence means restriction aims.

### 3.1. Presence of Others and Visibility

Previous work suggests that the presence of other people and potential visibility can be an important factor for some individuals in influencing decisions leading up to or during a suicide attempt. For example, the potential of being seen by other people may influence choice of location (Marsh et al., 2021; Marzano et al., 2019), and as such, they may avoid busy or monitored environments. Additionally, some individuals have indicated that the presence of other people has previously led them to disengage from pursuing an attempt (Marsh et al., 2021). From a goal-pursuit perspective, this might suggest a change to the expected outcome. One reason for this may be the potential negative impact on witnesses (Mackenzie et al., 2025; Marsh et al., 2021; Norman et al., 2023). However, some individuals have reported actively avoiding others or waiting for people to leave as a way of avoiding intervention (Mackenzie et al., 2018). This might suggest that encountering this unexpected development can lead individuals to re-assess the situation in ways that influence outcome expectancies and, in turn, behaviour (indeed, as a participant in one study described, the presence of another person “increases the chance of intervention from zero” (Mackenzie et al., 2025, p. 9)). Notably, re-assessment of beliefs does not necessarily require effortful deliberation and may occur automatically, sometimes even at an unconscious level (Sharot et al., 2023). For instance, if a person were to encounter a barrier to goal attainment, their beliefs about their ability to achieve the goal might change, even if they are not aware of having re-evaluated the situation. This process, known as belief updating, supports dynamic decision making in complex environments and, as such, could influence the perceived likelihood of experiencing an intervention in real time. Simply put, for those seeking to avoid preventive interventions, the presence of others may present a potential barrier to attainment that may lead them to update their beliefs around how likely it is that they will be able to proceed, which in turn may reduce motivation.

Determining how the presence of others might influence motivation may be useful for understanding behaviour. While factors such as proximity of others and ability to avoid them will, of course, be relevant, here we focus on the potential impact on the time gradient—specifically, whether the presence of others increases the salience of a potentially imminent intervention. In creating a perceived timeframe within which an individual must act to avoid intervention, the presence of others may lead some individuals to temporarily disengage from proceeding onwards. Indeed, individuals with lived experience of attempting suicide have reported that being spotted by another person can end a perceived opportunity, and that the presence of others can create a sense of pressure that feels off-putting (Marzano et al., 2019). This might suggest that, at least for some, time pressure produced by the presence of other people may make an action feel less feasible and therefore may act as a short-term deterrent. Yet, another possible outcome in such circumstances is that individuals may act more rapidly, such as refraining from pausing (e.g., Marsh et al., 2021), to avoid intervention.

#### 3.1.1. Lighting-Based Interventions

As we discuss in more detail further on in this section, how an individual responds to a potentially imminent intervention will likely be influenced by other factors (e.g., motivational value, proximity). However, if we assume that any perceived timeframe begins from the moment that an individual is noticed by another person, the anticipation of this may also motivate some individuals to avoid attention. For instance, they may seek out ways to be less visible to others, such as hiding before making an attempt (Mackenzie et al., 2018, 2025; Ryan, 2017). It might therefore be that one way to understand the effectiveness of measures intended to improve visibility within an environment is to consider how they might impact perceived timeframes for potential intervention and, in turn, motivation. For example, previous work suggests that installing blue lights at platform ends may be helpful for preventing suicides (Matsubayashi et al., 2013, 2014), although the extent of the effectiveness of this approach has been contested (Ichikawa et al., 2014). While some suggest this may be due to blue lights producing a “calming effect” on individuals, another pilot intervention at two station platforms in Sweden found that illuminating the ends of platforms with “blue lights” reduced the number of people willing to wait for their train in those areas (National Centre for Suicide Research and Prevention et al., 2022). Such a change in passenger behaviour is arguably difficult to explain through potential “calming effects”.

Whether blue light interventions impact motivational drive by influencing expectations about the likelihood of being seen in an environment remains unclear at present. However, the use of lighting as a way to encourage safe behaviour within rail environments has been tested in other ways (Ryan et al., 2022). Within the wider psychological literature, it has been found that the brightness of lighting can be associated with heightened levels of public self-awareness and the self-regulation of behaviour (Steidle & Werth, 2014). In other words, in brightly lit environments, individuals may be more aware of how they are viewed by others and might adapt their behaviour to blend in. From this perspective, a well-lit environment may influence prevention efforts if it increases expectations of being noticed by other people and, in turn, shifts the perceived likelihood of a potentially imminent third-party intervention (e.g., Mackenzie et al., 2025).

Why, then, when thinking about goal-directed motivation might we expect the use of “blue lights” to be any different from standard lighting? Blue-enriched lights (Chellappa et al., 2011) and bright white lights (Vandewalle et al., 2006) are both thought to increase levels of vigilance. It could be the case that by heightening an individual’s attention towards situational cues within the environment (e.g., those representing a risk of third-party intervention), blue lights influence perceptions of visibility in a similar way to bright white light. Indeed, research suggests the alerting effects of blue lights are independent of light intensity and instead may be the result of light wavelength (Beaven & Ekström, 2013). As such, a blue light may not need to be as bright as a white light for heightened alertness effects to occur.

Without further research it is not possible to determine the psychological processes that may underpin any potential preventative effect from blue light measures. However, given the ongoing interest in blue lights as a potential suicide prevention measure, whether the properties of blue light (e.g., its shorter wavelengths) make it an appropriate measure for influencing behaviour at high-risk locations is worth considering here. For example, previous work suggests that the influence of blue light on the alerting effect is thought to be mediated by eye colour (Beaven & Ekström, 2013). Specifically, its effects are potentially stronger for those with lighter-coloured irises. There is also evidence to suggest people with blue eyes may be more sensitive to the effects of light generally due to reduced melanin pigment increasing light transmittance (e.g., Nischler et al., 2013; Terman & Terman, 1999). While that is not to say that any influence of blue light on behaviour will necessarily be affected by person-level factors such as eye colour, it does highlight a need for further research outside of suicide prevention to be able to ensure its use would not disadvantage some groups over others (e.g., ethnic groups where darker eyes are a dominant trait) in this context.

#### 3.1.2. Presence of Surveillance Technologies

Given the environmental complexity and scale of some high-risk locations, one potentially useful consideration is whether the proposed “potentially imminent intervention” timeframe can better inform our understanding of how and when surveillance technologies may help prevent suicides. As discussed in the previous section, the perception of being visible to other people may be enough to influence behaviour. Research suggests this may also extend to some surveillance technologies. For example, previous work has found that some suicidal individuals may actively avoid security cameras (Marsh et al., 2021), while others have reported instances of individuals who moved to alternative locations after thinking they could be being watched through CCTV (Mackenzie et al., 2018). This might suggest that the “presence of others” does not need to be physical to be effective at influencing behaviour leading up to a suicide attempt but instead can be inferred in other ways (e.g., through the presence of a surveillance device).

**Figure 7:**
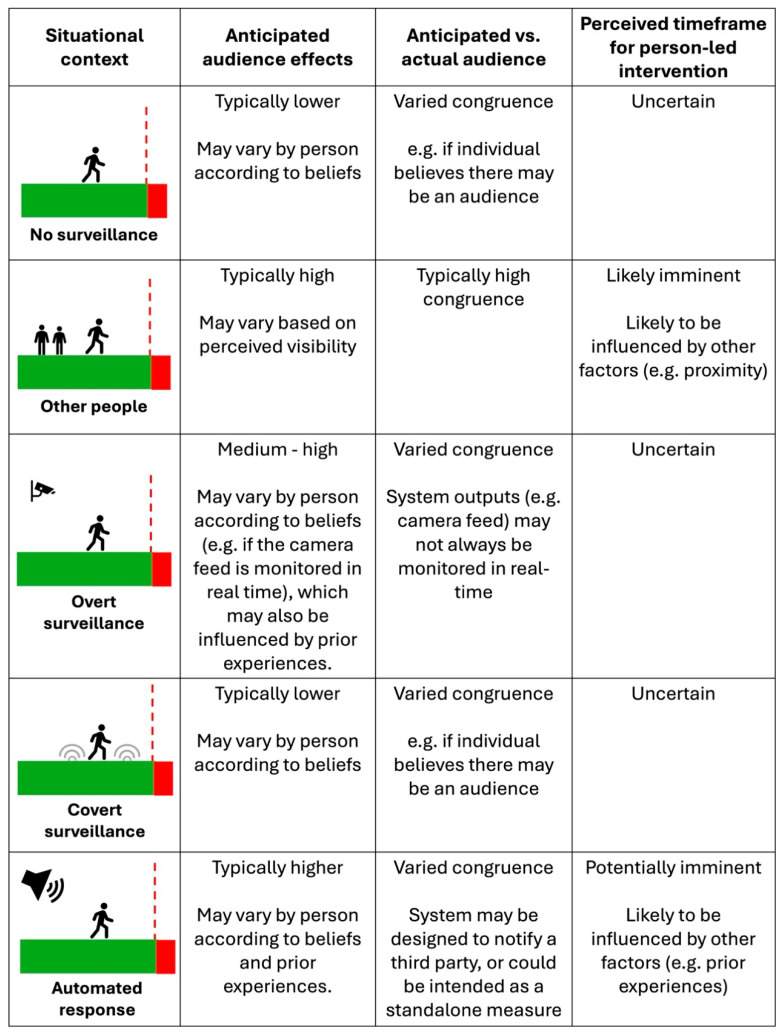
Summary of audience effects and perceived timelines for person-led intervention by surveillance type.

From a psychological perspective, the design of surveillance (and any associated processes) is likely to influence interpretations of intervention timeframes and likelihood in distinct ways (Figure 7). Firstly, if the surveillance is overt, then perceptions of being monitored are likely to be more salient than if covert. Put simply, the more visible and identifiable surveillance devices (e.g., cameras) are, the more aware individuals may be of potentially being under surveillance. As changes in behaviour can occur when an individual believes they are being observed by another person, i.e., “audience effects” (Hamilton & Lind, 2016), it is the perception of being under surveillance, rather than the reality, that is likely to inform outcome expectancies. Indeed, for certain types of technology, anticipated audience effects may not always correspond with actual audience effects (Kaye et al., 2022)—for example, if surveillance cameras are not actually being monitored in real time. This may be why one Australian study found that, after controlling for other factors, the number of cameras visibly installed in and around railway stations was associated with lower rates of suicide (Too et al., 2015). By increasing the salience of being monitored, identifiable devices (e.g., cameras) may indirectly increase the perceived likelihood of experiencing an intervention in ways that could influence motivation to proceed onwards for some individuals.

It could also be the case that the perception of a potential intervention within an uncertain timeframe may be enough to create doubts around proceeding onwards for some individuals. However, without the physical presence of other individuals, the timeline within which a potential intervention may occur is likely to be unclear (but may still be anticipated (e.g., Kaye et al., 2022)). While uncertainty of outcomes may create doubts around how best to proceed, the inability to gauge time remaining and proximity to a person-led response may lead an individual to rely instead on their beliefs, including their prior experiences. As demonstrated in trespass prevention research (da Silva et al., 2006), where overt surveillance technology does not initiate a physical response, its overall effectiveness may wane over time (potentially due to a lack of person-led response becoming known). Therefore, an individual’s beliefs about potential consequences may determine whether overt surveillance is an effective psychological deterrent for preventing suicides.

Where surveillance is covert and therefore not visible, it may only influence an individual’s behaviour if they anticipate its presence or where its presence becomes known (for example, if a person-led response arrives as a consequence of covert monitoring). Indeed, police officers interviewed in Ross et al.’s (2020) study indicated that after individuals become aware that they had been under surveillance during one suicide attempt at a particular cliff site, they may speed up any future attempts at the same location to avoid similar interventions. As such, perceived timeframes based on prior experiences may influence intervention avoidance behaviour in some circumstances, even without the physical presence of other people. This again illustrates how goal-directed motivation can be influenced by outcome expectancies (i.e., beliefs) rather than simply the information available to individuals in the immediate environment.

#### 3.1.3. Use of Automated Audible and Visual Deterrents

As well as the use of surveillance to promote a person-led response, some systems may produce automated announcements or initiate responsive lighting. While such systems have been trialled in the context of trespass prevention (e.g., da Silva et al., 2006; Kallberg & Silla, 2017), little is known about their effectiveness for suicide prevention. From a cyberpsychology perspective, the activation of these technologies may increase anticipation of a virtual audience due to the synchronous technological response to an individual’s behaviour (Kaye et al., 2022). If this also informs the anticipated response (e.g., the expectation that a person will also be notified), it may influence outcome expectancies of a likely intervention. In some cases, any sudden change in perception of an upcoming but uncertain deadline for avoiding intervention may be enough to create doubts about progressing onwards in the immediate moment.

However, concerns have also been raised about the suitability of these approaches for use in suicide prevention due to potential risks (Joyner et al., 2024). If, as we have previously suggested, perceiving a potentially imminent intervention influences motivational value via the time gradient, then there is a chance that this may lead some individuals to act more quickly in an attempt to avoid potential intervention. Indeed, as already noted, the goal-pursuit literature suggests goals become more motivating as the target becomes nearer (Ballard et al., 2022). This would suggest that the rapid presentation of cues signalling a potential intervention within an uncertain timeframe can be associated with a rise in motivational value for some people. Such a process may explain why previous research has shown that audible cues, such as fast train announcements and the sound of trains approaching, may trigger suicidal thoughts in some individuals (Mackenzie et al., 2025; Marzano et al., 2019), potentially even influencing some to speed up when walking towards a platform (Marsh et al., 2021). Others have reported that some individuals move to act quickly when they perceive they may experience an intervention (Ross et al., 2020). Individuals who are highly motivated to either avoid intervention or progress into a high-risk area may therefore be examples of those at particular risk.

**Figure 8:**
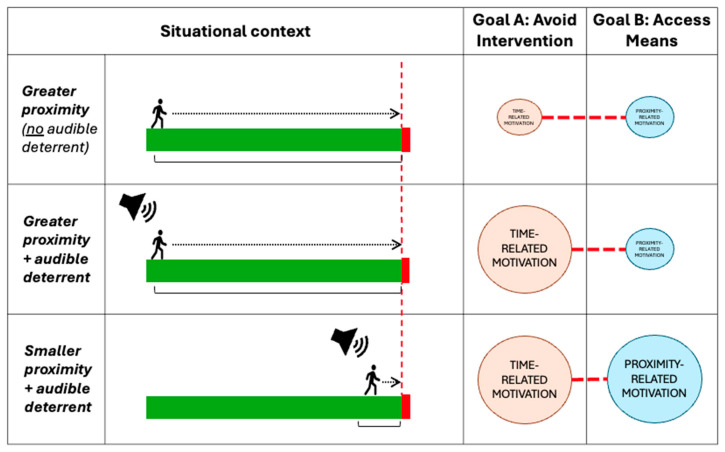
Combined influence of surveillance placement on attempt-related motivation.

In addition to potential intervention, another consideration that may shape an individual’s response to an automated surveillance system may be perceptions of associated timeframes. The goal-pursuit literature suggests that the perceived impact of one’s actions “are most salient when there is less time available” (Ballard et al., 2022, p. 168). This saliency of timeframes could help to explain, for example, the observed associations between shorter intervals between trains and the number of rail suicides (van Houwelingen et al., 2013). Moreover, the “deadline effect” is associated with a sensitivity to outcome probabilities, which may lead people to prioritise actions with certain outcomes over ones associated with uncertainty (e.g., proceed into a high-risk area vs. remain at the current position and risk potential intervention). The motivational values associated with any distinct but associated goals may therefore be important for understanding the overall drive to act (Figure 8). Although research is needed to determine whether this is the case, it may mean people could be more willing to take risks when an automated deterrent is triggered if their proximity to a high-risk area is relatively low (i.e., distance gradient), if the high-risk area is easy to access (i.e., rate-of-progress gradient), or if they perceive there to be a timebound opportunity in which to act (i.e., time gradient). As such, from a psychological perspective, the conditions around how an individual learns they are under surveillance (whether or not a human-led intervention will be coming in reality) has the potential to produce drastically different outcomes.

A further factor to consider here is the potential influence of individual differences—in particular, the ways in which a person updates their beliefs when presented with new information (e.g., an audible deterrent). While all of us can experience biases when updating our beliefs, some individuals may be more prone than others to experiencing challenges around the integration of new information. One example of this is the relatively common jumping-to-conclusion bias, which relates to a tendency to make hasty decisions based on insufficient information (Kube & Rozenkrantz, 2021) and which has been associated with factors such as cognitive reflection style, openness to the future (Peinado et al., 2024), and psychosis (e.g., Dudley et al., 2016; Rauschenberg et al., 2021). Research also suggests individuals more prone to jumping-to-conclusion bias may be more likely to rely on internal thoughts over contextual information when making these decisions (Özen-Akın & Cinan, 2022). This could mean that some individuals may be more likely than others to associate an audible deterrent with a potentially imminent intervention, without physical evidence that this would be the case (e.g., the presence of other people). While research is again needed to understand whether this would be the case, what is already known about the different ways in which people process information (especially under pressure) suggests that the potential impact of presenting an unexpected intervention (either physically or using technology) will likely differ across the population.

### 3.2. Drawing Attention to Atypical Behaviours

A final example of how suicide prevention measures may affect ability to avoid intervention is whether the measure can help draw attention to atypical behaviour. Previous work suggests that in the lead-up to a suicide attempt, some people may be motivated to behave in ways that they believe could (but may not actually) reduce attention from other people, e.g., “trying to look normal” (Mackenzie et al., 2018). Therefore, initiating behaviour that may be atypical within an environment (and therefore may draw attention) could create uncertainty for some. As previously discussed, attention from others may increase the salience of a potentially imminent intervention, and therefore anticipation of such attention may be demotivating for some people. This may help explain some of the psychological mechanisms underpinning some interventions.

Firstly, some measures may be designed to make atypical behaviours more prominent to others. An example of this is the use of lengthwise fencing at platform ends in railway stations. This design of fencing notably does not itself block access to the platform end. Instead, a transparent barrier is placed parallel to the platform edge, distinguishing those who stand in the space from others. Research suggests that such a measure may be useful for helping prevent trespassing at some locations, although the observed reduction in suicides was itself not significant (Fredin-Knutzén et al., 2024). It may be the case that when evaluating whether to enter some demarcated spaces, the potential for being noticed becomes more salient and as such presents a deterrent. However, another consideration is whether any time-related impact indirectly influences feasibility in a way that such a measure may be more effective in certain environments. Specifically, for example, in rail and metro environments, coordinating both the timings around avoiding an intervention in addition to the arrival of any train may decrease perceived feasibility of accessing the means for a suicidal act. Therefore, this may increase the likelihood of human-led interventions by drawing attention before a train arrives. Alternatively, the perceived complexity may be demotivating and encourage an individual to move elsewhere. Notably, both outcomes were observed in a study by Fredin-Knutzén et al. (2024). How perceptions of intervention timeframes impact the effectiveness of other measures is therefore a potentially valuable avenue for future research.

Indeed, motivations to avoid interventions may arguably also influence the effectiveness of means restriction measures. Behaviours such as climbing a barrier or standing in a high-risk environment would often be considered atypical and therefore may draw attention. The combination of a barrier both delaying efforts to access high-risk environments and helping draw attention to such efforts can create additional time that can facilitate the arrival of a third-party intervention (e.g., Bennewith et al., 2011). However, where an individual intends to proceed past a means restriction measure into a high-risk area, attention from others may also increase the perceived (not just the actual) likelihood that an intervention could happen. As the GOAL Architecture framework outlines, “rate of progress” is influenced by perceived time remaining (Ballard et al., 2022), and measures that increase the likelihood of a third-party intervention (e.g., surveillance) may impact overall outcome expectancies. Therefore, initiating atypical behaviours may indirectly threaten the perceived feasibility of accessing the area successfully (and the anticipation of which may reduce motivation to proceed).

Both of these perspectives provide examples for why considering how a measure might influence motivation may be helpful for determining any impact of combining different suicide prevention measures. While surveillance itself does not necessarily make it “physically harder” to access a high-risk area, whether it is effective at helping to prevent suicides when combined with means restriction measures is still to be determined. Moreover, measures that inadvertently produce some atypical behaviours (e.g., climbing a barrier) could be more effective if the actions are likely to be observed (e.g., via surveillance), but potentially less effective in a remote, unmonitored area. Understanding whether combined measures lead to an increase in rescues or an overall reduction in attempts may be helpful for determining how and why such measures are effective. Utilising a framework such as GOAL Architecture may be one way of quantifying the environmental changes.

## 4. Help-Seeking

While a person may proceed towards a high-risk location with the primary intention of accessing an area of danger, they may also hold goals around seeking help, even if these are associated with lower motivational value or are even inhibited. If that is the case, then the psychological mechanisms behind effective engagement with measures related to help-seeking will likely differ. In the following sections, the impact of help-seeking as an alternative goal is discussed before considering how applying the gradients from the GOAL Architecture framework may allow us to better understand willingness to engage in help-seeking behaviours.

### 4.1. Help-Seeking as an Alternative and/or Additional Goal

For some individuals, help-seeking may be an alternative and/or additional goal (one that is potentially inhibited) that conflicts with (rather than facilitates) the primary goal under pursuit when arriving at a high-risk location. As previously noted, some individuals may disengage from attempt-related goals through dynamic changes to their motivational value. This may present an opportunity for help-seeking goals to be pursued if the situation allows for it, even if the help-seeking goals were not prioritised upon arrival. However, for others, commitment to the pursuit of an attempt-related goal may be so strong that alternative outcomes are inhibited.

#### 4.1.1. Goal Shielding

As previously discussed, when an individual is highly committed to a “focal goal”, alternative goals that do not aid the attainment of the focal goal may be cognitively inhibited (Shah et al., 2002). While this “goal shielding” process may be beneficial in other contexts, it is likely to be detrimental if an individual’s focal goal is something that may cause harm. Indeed, Kopetz and Orehek (2015) suggest that for risky behaviours, goal shielding can allow alternative concerns to become automatically inhibited and lead to motivated distortion, where any consequences are distorted in line with the goal under pursuit. This may mean that for highly committed individuals, other options (e.g., help-seeking) or potentially even the implication of their actions (e.g., on others) may be inhibited. Simply put, someone who might consider seeking help in other contexts may not when highly motivated to pursue a suicide-related act.

The potential implications of goal-shielding are likely to be particularly relevant for understanding the psychological processes around goal pursuit for those experiencing psychological distress. Indeed, research suggests that people may be more likely to inhibit alternative goals when experiencing greater levels of anxiety (Shah et al., 2002) or when under acute psychosocial stress (Plessow et al., 2011). Similarly, affective states high in motivational intensity (e.g., fear) may narrow cognitive scope in ways that can hinder the processing of peripheral information (see Harmon-Jones et al., 2012). As these processes allow attentional resources to be allocated to a focal goal, they may therefore be particularly relevant when considering the effectiveness of help-seeking-related measures, such as signage encouraging help-seeking from a helpline, for example.

##### 4.1.2. “Bursting the Bubble”

Interviews with people with lived experience of surviving a suicide attempt suggest that in some cases external stimuli can potentially disrupt these focused states—for example, a child screaming (Geerling et al., 2019; Marsh et al., 2021) or haptic cues from digital devices (Heesen et al., 2024). From a psychological perspective, novel sensory changes within an environment may sometimes hinder behavioural performance, such as the introduction of auditory (SanMiguel et al., 2010) or tactile (Parmentier et al., 2011b) novel stimuli. Research suggests distraction may be more likely to occur when the stimulus is unexpected rather than predictable (Parmentier et al., 2011a). This might suggest that distraction from focused states may be more likely to occur when the stimulus is not anticipated during goal pursuit. Such a process may be one explanation for the “bursting the bubble” effect and may provide some insights into the conditions needed to help an individual disengage from an attempt-related goal.

Furthermore, given the previously noted concerns that unexpected stimuli (e.g., audible deterrents) may impact behaviour in opposing ways, it is important to highlight how “distracting” stimuli may not always produce the same outcomes. Indeed, while novel stimuli may distract in some contexts, they can facilitate goal pursuit in others—for example, when they inform individuals about the timing of a target’s arrival (see Schomaker & Meeter, 2015). This may suggest that, in some contexts, novel stimuli may provide information that can help an individual pursue their intended goal (e.g., audible deterrents alerting to a potentially imminent intervention). Recent research also indicates that the relevance of stimuli to primary and secondary tasks may influence whether it is perceived as distracting (Mandal et al., 2024), and therefore, unexpected stimuli may affect behaviour in some but not all situations.

With this in mind, it may be that some “unexpected stimuli” identified in previous interviews (e.g., baby crying, haptic alert) increase the salience of alternative social goals in ways that are sometimes (but potentially not always) helpful in the context of suicide prevention. For example, research suggests photographs of family may create feelings that conflict with any attempt-related goal, which could encourage some individuals to disengage from an attempt (Owens et al., 2019; Stevenson, 2016). However, depending on the circumstances surrounding an attempt, reminders of specific people may not be helpful (e.g., Lin et al., 2009). Moreover, while sounds of babies crying may disrupt focused states for some (Geerling et al., 2019; Marsh et al., 2021), for others it may exacerbate an existing situation, such as for women with perinatal mental health problems (Gair, 2008; Geerling et al., 2019). Therefore, while approaches to help “burst the bubble” may be a useful strategy for helping prevent suicides, the design of any such interventions would still need to be carefully considered.

### 4.2. Contextualising Help-Seeking at a High-Risk Location

There is no single reason why an individual may wish to seek help while at a high-risk location. For example, they may have disengaged from an attempt-related goal in ways that allow help-seeking-related goals to become salient. Others may approach a high-risk location with the intention of leaving outcomes up to fate (Marsh et al., 2021), which for some might mean they may be open to engaging in help-seeking if the right opportunity were to present itself. Alternatively, it has been suggested that in some instances suicide attempts may have a communicative function—for example, to signal distress or a desire to seek help (May et al., 2020). Moreover, while potentially less common, these “communication motivations” have also been associated with reduced intent and increased probability of intervention (May & Klonsky, 2013). As such, underlying reasons for pursuing a suicide attempt may also influence willingness to engage with help-seeking opportunities. However, regardless of whether an individual has gone to a location to seek help, or if they happen to be at the location when they become motivated to seek help, it may be beneficial to consider what is known about help-seeking behaviours in relation to gradients identified in GOAL Architecture.

Measures that enable help-seeking at high-risk locations may be implemented in a variety of ways. In some cases, there might be people based at the location—for example, staff who have completed gatekeeper training who may be able to facilitate referral to appropriate support (Burnette et al., 2015). Other measures may encourage or mediate help-seeking without the physical presence of a person—for example, installing signage offering support and identifying routes to access help (e.g., helpline signage placed at the location) or placing technology to mediate communication between an individual and remote support (e.g., crisis phones). From a proximity perspective, it may be the case that the location is relatively closer and/or easier to reach than other locations associated with potential support.

If that were the case, then the distance-related motivational value of pursuing help-seeking should be greatest for locations that are most accessible to reach. Indeed, evidence from the wider suicide prevention literature supports this idea. For example, Tadmon and Bearman (2023) found that the suicide risk of individuals was greater the further away they were from accessing mental health care. Similarly, a recent study found men may be at increased risk of dying by suicide as travel time to getting care from the nearest general or psychiatric hospital increases (Barry et al., 2023). Previous work also found that living distance from school was positively associated with increased suicidal ideation in male students (Armstrong & Manion, 2006), suggesting the influence of “distance” is not only associated with formal sources of help. While distance to support is often likely to be reflective of wider systemic issues and other potential co-founders (e.g., distance from hospital in relation to rurality), the general concept of “proximity” to help may also be useful for developing effective ways to tackle these wider issues. For example, a study found providing veterans located in rural areas with a video-enabled tablet helped to increase their levels of engagement with mental health care (e.g., psychotherapy sessions) and was associated with a reduction in suicide behaviours (Gujral et al., 2022). However, these examples illustrate why it may be beneficial to consider the role of distance as a factor potentially informing choice of help-seeking location, as well as to understand the most effective ways to engage those who may be motivated to seek help.

Furthermore, understanding the wider societal context in relation to the time gradient may be a potentially important consideration both for those who are or become motivated to seek help, and those for whom a waning motivation to pursue a suicide attempt may present an opportunity for prevention efforts encouraging help-seeking. Indeed, delays and long wait times for support may lead some individuals to perceive that they may never receive help, which research suggests may be a consideration leading up to an attempt (Punton et al., 2022). This ties into the importance of wider availability and accessibility of mental health support beyond the high-risk location. Others have found long wait lists can also present a barrier for accessing formal support (e.g., Heinig et al., 2021; Wilson & Marshall, 2010), demonstrating the importance of facilitating provision of help when requested. Arguably, some of the factors relating to why someone may be, or become, motivated to seek help while at a high-risk location will be beyond the control of those who oversee the site. However, the dynamic nature of goal pursuit highlights the potential need to encourage engagement with help-seeking goals as, if disengaged from these, potentially harmful alternatives may instead be prioritised.

#### Effectiveness of Help-Seeking Measures from a Goal-Pursuit Perspective

If, as proposed by GOAL Architecture (Ballard et al., 2022), motivation to pursue help-seeking actions is influenced by time, proximity, and perceived ease, then it may also be valuable to consider how help-seeking measures relate to these gradients, both for evaluation purposes and for supporting the development of effective initiatives. Openly providing individuals with relevant information may be one example that could be beneficial for increasing associated motivational value. For example, advertising phone numbers for helplines may remove the need to research or ask for such information, arguably reducing the progress required to access help (i.e., distance gradient), which in turn may increase the motivational value of help-seeking goals. Furthermore, effective messaging may help destigmatise help-seeking (i.e., rate-of-progress gradient) and potentially encourage individuals in ways that may influence motivation to act (e.g., Duthie et al., 2024). Such measures do appear to influence help-seeking behaviour. Research from the rail industry suggests that running advertising campaigns with local suicide prevention helplines has been associated with increased rates of calls (Gabree et al., 2019). Another study found that over the course of 16 months, signage placed at platform ends at a railway station in Denmark encouraged at least 13 people to call a suicide prevention helpline (Erlangsen et al., 2023). As these examples illustrate, evaluating help-seeking measures using outcomes related to the targeted goal (e.g., number of calls made) may be useful for understanding their effectiveness.

In turn, considering the design elements of help-seeking measures in line with gradients outlined in GOAL Architecture may help optimise their effectiveness. For example, by removing some of the additional barriers individuals may encounter contacting a helpline (e.g., finding a phone or mobile signal, memorising and entering numbers), it may be that the addition of crisis telephones or call help points may reduce some of the potential difficulties around making a call. Such resources do appear to be used for help-seeking (Erlangsen et al., 2023) even if their overall effectiveness for suicide prevention is somewhat mixed (see Pirkis et al., 2015). However, previous work has identified the importance of being able to discretely engage in help-seeking behaviours at public locations (Mackenzie et al., 2025). As such, whether overtly labelled help points could increase perceived likelihood of being noticed by passers-by may be another important design consideration. In contrast to other types of suicide prevention measures, ensuring that interventions to support help-seeking quickly facilitate accessible and/or nearby support may be some of the ways in which they could help encourage help-seeking behaviours.

A further consideration is whether it is possible for some measures that enable help-seeking to be developed in ways that also challenge beliefs related to attempt-related goals in an appropriate manner. Indeed, research by Owens et al. (2019) suggests that during a person-led intervention, caring gestures and (permitted) touch may be useful for tackling feelings of isolation. Others have suggested that offering practical support to confront and address specific stressors may be helpful for some people to regain some sense of control (Player et al., 2015). While signposting to support may also be useful for improving perceived proximity and/or rate of progress towards addressing stressors, considering ways in which this could be tailored to specific needs may be especially important from a goal-pursuit perspective. Indeed, previous research with farmers suggest that they may not always feel that support available to the general public will be useful, especially if they anticipate that these those manning these services may not have a good understanding of their specific needs (Hagen et al., 2022). The ability to signpost to tailored support where possible—for example, by making staff aware of official databases of support organisations that can be filtered by specific need (e.g., publicly accessible websites such as Hub of Hope (https://hubofhope.co.uk/) and Find a Helpline (https://findahelpline.com/))—could be one way of helping to reduce steps to access relevant support. While these approaches will not work for everyone, understanding how existing help-seeking measures (e.g., communications) may help to facilitate or challenge specific goals may be a useful step for understanding when they may be most effective.

Finally, identifying potential barriers to attaining help-seeking goals is likely to be important for developing effective measures. An individual may be willing to pursue a help-seeking goal but may not be able to access the offered support. For example, they may encounter a sign encouraging them to approach members of staff for help but find themselves unable to locate anyone (Marsh et al., 2021). By increasing the effort required to find help, this may reduce motivation via the rate-of-progress gradient. Alternatively, help may not arrive within an anticipated timeframe, creating uncertainty around waiting times that could be demotivating and risk reinforcing feelings of hopelessness for some individuals (Punton et al., 2022). Ensuring offers of help are fulfilled may arguably be important for these measures to be effective. Furthermore, concerns about the consequences of engaging in help-seeking—for example, due to prior negative experiences (Holt et al., 2024) or uncertainty of what might happen (Marsh et al., 2021)—may also influence motivation to engage in help-seeking behaviour generally. For others, uncertainty around potential costs that might arise after accessing support may amplify existing financial concerns (Hagen et al., 2022). Clear and transparent communication where appropriate may therefore be helpful for ensuring accurate outcome expectations. Together, these examples illustrate the importance of carefully designed processes around help-seeking measures at high-risk locations to ensure their effectiveness.

## 5. Discussion

In this article, we have sought to explore the potential ways suicide prevention measures implemented at high-risk locations could influence levels of goal-directed motivation based on the principles outlined in GOAL Architecture (Ballard et al., 2022). Specifically, by identifying how they may impact perceptions of distance, time remaining, and rate of progress, we have considered how these measures could impact motivational drive associated with attempt-related actions, in terms of both prevention and potential risks. We began by exploring the ways in which measures that only partially restrict access to means of suicide may shift the motivational value associated with related goals. First, we discussed the potential role of half-height barriers and other physical adaptations (e.g., spinning bars) in reducing perceived ease of access (while not making access impossible). We then proposed that previously observed psychological deterrent effects may be in part due to the impact of partial measures on perceived rate of progress, which in turn may influence motivational drive to proceed past them. We also described how person-level factors, including self-efficacy and appraisals of risks, may influence the strength of any psychological deterrent effect of measures that partially restrict access to means, which could in turn impact the effectiveness of the measures for certain groups of people. The impact of measure design on perceived distance to suicide means was also discussed in the context of measure placement and proximity of alternative access points.

Next, we proposed that perceptions of being monitored may create a timeframe within which an individual believes they can act, which may in turn influence levels of motivation to avoid a third-party intervention. We firstly considered how the presence of others may influence expectancies of a potentially imminent intervention, and discussed the role that lighting-based interventions (e.g., blue lights) may play in informing perceived visibility. We then explored the impact that different types of surveillance technologies (e.g., covert, overt) may have on perceptions of monitoring and timeframes for third-party intervention. The potential implications of this for the use of automated systems (e.g., that emit an audible deterrent upon activation) as a suicide prevention measure was also discussed. Moreover, the ability of some measures to help draw attention to atypical behaviours, potentially influencing perceived feasibility (via changes to rate of progress), were considered.

Finally, we looked at help-seeking as an alternative and/or additional goal that for some may exist alongside a primary goal (e.g., “accessing means of suicide”) and could at times be inhibited (e.g., due to goal-shielding processes). The implications of goal-shielding on the effectiveness of measures to enable help-seeking was discussed, as well as the potential role of external stimuli for disrupting these states of focused goal pursuit (e.g., “bursting the bubble”). We also applied the gradients identified by the GOAL Architecture framework (Ballard et al., 2022) to contextualise the role of help-seeking measures at high-risk locations and consider how the framework may be applied to aid the evaluation and development of these measures.

Many suicide prevention measures implemented at high-risk locations will to some extent be reliant on psychological mechanisms for their effectiveness, whether they encourage disengagement from an attempt-related goal or promote engagement with actions like help-seeking. Alternatively, others may indirectly influence goal pursuit (e.g., by increasing perceptions of a potentially imminent third-party intervention). Through examining the existing literature on suicide prevention at high-risk locations with a goal-pursuit lens, we have sought to identify potential psychological mechanisms that may help explain these effects. We hope that our formulation adds to previous research that has considered suicidal and self-injurious behaviours in the context of goal-directed processes (e.g., Kopetz et al., 2018; Valach et al., 2006, 2016). However, we have also discussed the potential influence of encountering suicide prevention measures on the pursuit of subordinate-level goals in a way that allowed us to not only consider the extent to which a measure may be effective at preventing suicides but also to identify possibilities for increased harm.

### Limitations and Opportunities

There are, of course, limitations to the approach taken here. By integrating theories of goal pursuit, GOAL Architecture provides a descriptive framework to quantify the influence of changes to goal attainability on motivation (Ballard et al., 2022). Considering how goal dynamics may play out in relation to gradients of distance, time remaining, and rate of progress can be a useful exercise for identifying future areas of investigation, as we have attempted to do here. However, alternative approaches may also provide useful insights in this area, such as situated or embodied cognition. Future work may therefore approach this issue from different perspectives.

Moreover, the extent to which suicide prevention measures have the potential to adjust attempt-related motivational drive should not be overestimated. Firstly, the characteristics of any corresponding, higher-level goals (e.g., approach vs. avoid) will also determine whether motivation increases or declines as goal pursuit unfolds. As discussed here in the context of means restriction measures and proximity, the effectiveness of a measure may be somewhat dependent on the types of higher-level goals involved. Overall levels of desire are likely to play a central role in determining whether any measure is likely to influence behaviour.

From this perspective, the psychological effects of encountering a suicide prevention measure would be additive and therefore determined by existing motivational states. As such, it is not our intention to dismiss the importance of other sources of motivation potentially driving an attempt, but rather to consider how suicide prevention measures may further influence attempt-related decisions and behaviour. For example, the model proposed in the Theory of Interpersonal Behaviour identifies “facilitating conditions” as a moderator between two factors (“habits” and “intentions”; i.e., attitudes, social factors, and affect) and behaviour (Triandis, 1977). In previous studies, “facilitating conditions” has been represented by factors such as time constraints (Kupfer et al., 2019), perceived difficulty/ease of an act, and walking distance (Osei et al., 2024). Therefore, the framework proposed in GOAL Architecture may be a useful way of identifying ways of quantifying the influence of suicide prevention measures so that any effects may be appropriately accounted for in other models of behaviour.

Moreover, we have focused on three recommended categories of suicide prevention measures for high-risk locations and how they may impact judgements and behaviour relating to specific attempt-related goals within these environments. This adds to previous work that has considered the potential role of suicide prevention measures at high-risk locations at different stages of the suicidal process (Mackenzie et al., 2025) and in relation to events leading up to an attempt (Burkhardt et al., 2014). By focusing on a relatively small number of subordinate goals (i.e., accessing means, avoiding third-party intervention, help-seeking), we do not address the periods leading up to and after an attempt, or the wider dynamic network of goals (including higher-level goals related to the attempt and conflicting goals). Such a granular approach, we believe, may be a necessary step in furthering our understanding of how, why, and when certain measures are (or are not) effective at preventing suicides in these environments.

This is particularly important when thinking about the range of factors that may influence suicidal behaviour and, in particular, suicide attempts. Focusing on any impact on goal pursuit as it unfurls may be useful for understanding the role prevention measures play within high-risk environments at specific points in time, as well as acknowledging their limits. For example, while an individual may disengage from an “access means” goal at a specific location (e.g., subordinate goal), it does not mean the overall motivation to pursue a suicide attempt has reduced or ceased. While for some individuals, disengagement from accessing means at one location may be enough to protect them in the immediate (but not necessarily longer) term, for others there may be a risk of displacement. Alternatively, an individual may engage in help-seeking behaviours but then encounter barriers to accessing help (e.g., busy phone lines). From this perspective, being motivated to engage in behaviours will not necessarily result in goal attainment. These kinds of considerations are arguably important when designing safe environments and effective processes and therefore warrant further attention; however, they are not long-term solutions when it comes to helping an individual.

We hope that our detailed consideration of the psychological processes associated with suicidal behaviour at high-risk locations will prompt further thinking about the complex interplay of factors that may influence suicidal behaviour and its prevention in these and other situations. As others have previously noted (e.g., Kopetz et al., 2018), examining complex behaviours through a goal-pursuit lens can help us develop a deeper understanding of them. It may also therefore be beneficial for those designing suicide prevention measures more generally to consider how principles of goal pursuit and goal prioritisation could be applied in a similar manner—for example, to understand different types of experiences, such as feelings of ambivalence or distress presentations. Applying frameworks like GOAL Architecture (Ballard et al., 2022) to explore concepts of dynamic goal prioritisation at various stages of the suicidal process may be one approach that could prove useful for identifying areas of opportunity for suicide prevention.

## Data Availability

No new data were created or analysed in this study. Data sharing is not applicable to this article.
